# Comparative efficacy of perioperative lidocaine infusion versus thoracic epidural analgesia for pain management in abdominal surgery: systematic review and meta-analysis

**DOI:** 10.1016/j.bjane.2025.844616

**Published:** 2025-03-29

**Authors:** Gustavo R.M. Wegner, Bruno F.M. Wegner, Ramon Huntermann, Manoela L. Pinto, Júlia A.P. Vieira, Amanda P. de Souza, Francisco J.L. Bezerra

**Affiliations:** a Universidade Federal da Fronteira Sul, Passo Fundo, RS, Brazil; b Universidade Federal do Rio Grande do Sul, Porto Alegre, RS, Brazil; c Centro Universitário para o Desenvolvimento do Alto Vale do Itajaí, Rio do Sul, SC, Brazil; d Universidade Federal de Ciências da Saúde de Porto Alegre, Porto Alegre, RS, Brazil; e Universidade da Região de Joinville, Joinville, SC, Brazil; f Universidade Federal de Juiz de Fora, Juiz de Fora, MG, Brazil; g Rede D'Or São Paulo, São Paulo, SP, Brazil

**Keywords:** Analgesia, Epidural analgesia, Lidocaine, Meta-analysis

## Abstract

**Background:**

Recent randomized clinical trials have compared the perioperative use of Intravenous (IV) lidocaine and Thoracic Epidural Analgesia (TEA) for postoperative analgesia in patients undergoing abdominal surgery.

**Methods:**

A systematic search was conducted on Embase, Web of Science (all databases), Cochrane Library, and PubMed on March 25, 2024, adhering to the Cochrane Handbook and PRISMA guidelines.

**Results:**

Out of 1261 screened studies, 6 were included. TEA provided superior pain relief on a 0 to 10 pain scale at rest compared to IV lidocaine at 2 (n = 335, MD = -0.72, 95% CI -0.19 to -1.25, p = 0.007423, I^2^ = 83%) and 24 hours postoperatively (n = 402; MD = -0.18, 95% CI -0.12 to -0.23; p < 0.000001, I^2^ = 18%). However, no statistically significant differences were observed on pain scores at rest at 48 and 72 hours. TEA provided superior pain relief on a 0 to 10 pain scale during coughing at 24 hours postoperatively (n = 360; MD = -0.36, 95% CI -0.19 to -0.52, p = 0.000019, I^2^ = 2%), but no statistically significant differences were observed in pain scores on coughing at 48 and 72 hours. There were no statistically significant differences in postoperative nausea and vomiting, time to first flatus, or length of hospital stay.

**Conclusions:**

TEA provides more effective postoperative pain relief compared to IV lidocaine during the first postoperative day, as evidenced by analyses of pain both at rest and during coughing.

## Introduction

Postoperative analgesia in abdominal surgery has been a subject of debate. Although Thoracic Epidural Analgesia (TEA) is considered the gold standard for such procedures, several considerations regarding its use remain. Factors such as the availability and the ease of administration of the analgesic technique must be weighed against potential failure rates and complications associated with the chosen method. Moreover, the selected technique should align with the patient's specific pain management needs. In this context, one important discussion point concerns the application of TEA in laparoscopic surgeries, where there is uncertainty regarding its clinical superiority over alternative approaches for postoperative analgesia.^1^, ^2^, ^3^

Adding to the uncertainty surrounding the clinical superiority of TEA, recent studies have explored the potential use of perioperative lidocaine infusion as a viable alternative for abdominal surgeries, as it offers ease of administration and broader availability. The debate surrounding this issue has been reinforced by the recent publication of a randomized clinical trial suggesting the non-inferiority of perioperative intravenous lidocaine infusion compared to thoracic epidural analgesia in major abdominal surgeries.^4^

A previous meta-analysis conducted by Weibel et al., which included 102 patients from two randomized clinical trials, assessed pain at 24 and 48 hours postoperatively and the time to the first bowel movement, but found no statistically significant differences in any of the outcomes analyzed.^5^ However, their analysis was limited by the inclusion of only two studies, which considerably weakened the robustness of their conclusions.

With the emergence of new evidence, our meta-analysis seeks to expand the previous analysis by leveraging greater statistical power to evaluate whether the analgesic effect of lidocaine is comparable to that of TEA and to determine whether perioperative lidocaine infusion could serve as a viable alternative to TEA for abdominal surgeries.

## Methodology

The current systematic review explores the efficacy of perioperative lidocaine infusion compared to thoracic epidural analgesia in managing postoperative pain in abdominal surgeries. The methodology adheres to the guidelines outlined in the Cochrane Handbook and follows the criteria recommended by PRISMA (Preferred Reporting Items for Systematic Reviews and Meta-Analyses).^6^^,^^7^ We followed the recommendations of the D'Souza et al. guideline for the presentation of our results.^8^

### Registration

PROSPERO ID: CRD42024528707.

### Eligibility criteria

The inclusion criteria followed the Population-Intervention-Comparison-Outcome (PICO) principle, as follows: adult patients undergoing abdominal surgeries (P), perioperative lidocaine infusion (I), thoracic epidural analgesia (C), and pain scores assessment (O). There were no restrictions on including articles that presented non-inferiority methodology. There was no distinction in the inclusion between studies that performed thoracic epidural analgesia based on landmarks or based on fluoroscopy.

The exclusion criteria encompassed studies that were not randomized clinical trials, involved patients undergoing non-abdominal surgeries, employed lumbar epidural analgesia or regional anesthesia techniques other than thoracic epidural analgesia, inadequately described the epidural analgesia technique used, failed to administer intravenous lidocaine infusion, or did not report pain score outcomes.

### Search strategy

The search was conducted on electronic search engines: PubMed, Embase, Cochrane Library and Web of Science (all databases). Identified study protocols were checked for results. The reference lists of all the included articles were also reviewed for potential citation eligibility. There was no restriction regarding language or publication date.

The full search strategy for all databases is available in our Supplementary Material. The searches were conducted on March 25, 2024. A new search was conducted before the submission to the journal, with no new studies within our eligibility criteria identified.

The identified documents were exported to a reference manager (Rayyan) to remove duplicates.^9^ Two independent reviewers (GRMW and RH) conducted a two-step selection process. Initially, studies were screened based on titles and abstracts, followed by a thorough review of the full texts of the articles selected in the initial step. In cases of disagreement, a third reviewer was consulted (FJLB).

### Data extraction and synthesis

Parallel and independent duplicate data extraction was conducted using standardized spreadsheets in Google Sheets. This process covered various aspects including article identification, sample sizes, age, sex, type of surgery, ASA classification, and outcome measures such as pain scores at rest and on coughing at 1‒4 hours postoperative, as well as at the 12-, 24-, 48-, and 72-hour marks postoperative. Inpatient time, time to first flatus, time to first bowel movement, time to advancement to clear liquid diet and PONV were also extracted.

Subsequently, the relevant data was organized by creating new tables to improve comprehensibility. When data was described as recorded but inaccessible, we reached out to the corresponding author for data retrieval. For continuous data extracted from studies that only provided sample medians and ranges or first and third quartiles, calculations were used to estimate the sample mean and standard deviation.^10^^,^^11^ For data presented solely as images, WebPlotDigitizer 4.7 was utilized to extract the relevant data.^12^

We contacted the corresponding authors of the studies by Wongyingsinn et al.^13^ and Jayaprabhu et al.^14^ by email to clarify the information presented in the articles, but we did not receive a response.

### Outcomes assessment

Our primary outcomes were the postoperative pain scores. Other outcomes were considered secondary outcomes in our analysis. To maintain the reliability and robustness of the results and in accordance with the Cochrane Handbook, only outcomes with three or more studies were included in the analysis.

We assessed the quality of evidence for our primary outcomes in duplicate using the Grading of Recommendations Assessments, Development, and Evaluation (GRADE).^15^

### Meta-analysis

We opted to utilize software R Studio and the meta package v4 for conducting the meta-analyses to align with the guidelines proposed by D'Souza et al.^8^ Odds Ratio (OR) with 95% Confidence Intervals (95% CI) was used to compare treatment effects for categorical endpoints. The Mantel-Haenszel (MH) method was used for the analysis. Mean Difference (MD) with 95% CI was used for continuous endpoints. Significant heterogeneity was considered with I^2^ > 40%, and statistical significance was set at p < 0.05, as suggested by the Cochrane Handbook, chapter 10.10.2.^6^ Heterogeneity was assessed using the Restricted Maximum Likelihood (REML) method. A random-effects model was used for analysis. *R* software version 4.4.0 was used for statistical analysis.

Prediction intervals were used to better assess the precision of the estimates. A prediction interval provides a range within which we expect a future observation to fall with a certain level of confidence. While prediction intervals are not directly used in the Grading of Recommendations, Assessment, Development, and Evaluation (GRADE) approach to assess the quality of evidence, they can indirectly inform judgments about precision, which is one of the factors considered in GRADE assessments.

Additionally, we present heterogeneity along with the confidence interval for heterogeneity, which further enhances the reliability of the results in the GRADE assessment.^8^

### Leave-one-out sensitivity analysis

We conducted a leave-one-out analysis for outcomes with three or more studies to assess the robustness of the findings. Changes in the significance of heterogeneity or shifts in the direction of results were considered significant alterations and described in the results. If there were no changes in the significance of heterogeneity or shifts in the direction of results, we described the outcome as consistent and not dependent on individual studies.

### Trial sequential analysis

A trial sequential analysis was conducted to assess the consistency of the outcomes analyzed, with parameters set at a type 1 error of 5% and a type 2 error of 20%.

### Risk of bias assessment

For randomized clinical studies, the Revised Cochrane Risk-of-Bias Tool for Randomized trial (RoB2) was used.^16^

## Results

### Study selection and characteristics

**Figure 1:**
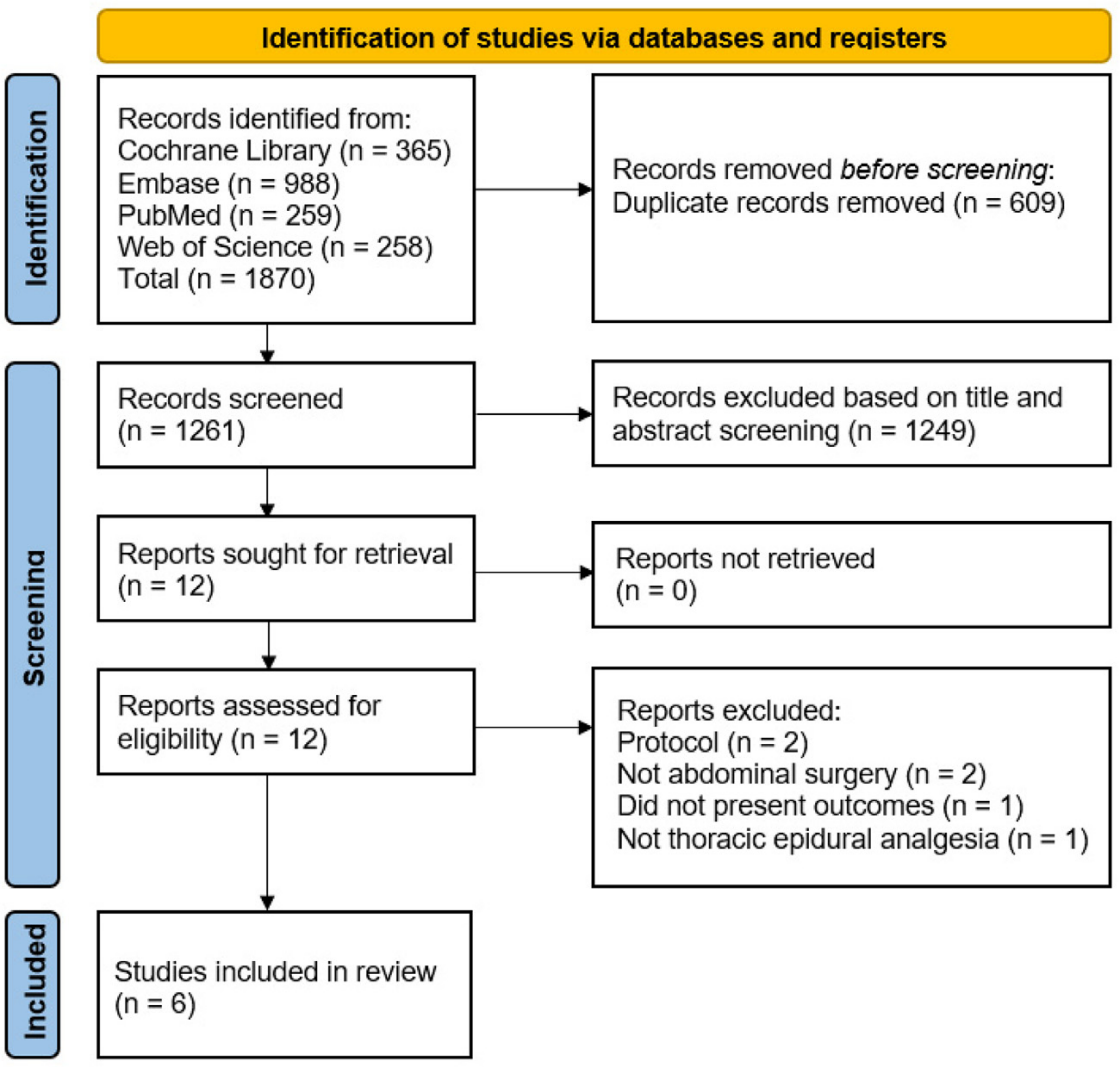
PRISMA flowchart.

We screened 1261 manuscripts, as shown in the PRISMA flowchart (Fig. 1), and included 6 RCTs with 437 patients.^4^^,^^13^^,^^14^^,^^17^, ^18^, ^19^

Baseline characteristics are summarized in Table 1 (Table 1). Details on patient-controlled analgesia are available in Supplementary Table 1.

**Table 1:** Baseline characteristics of the RCTs included.

Study	Interventions			PCA/ PCEA	n	Age	ASA	Male (%)	Type of surgery
	Dose	Start	Time of infusion						
Swenson et al. 2010	TEA: Placed between the 8‒12^th^ T. ‒ BUPI 0.125% + HM 6 mcg.mL^-1^ at 10 mL.hr^-1^; dose adjusted post-surgery.	Within 1-hour postoperatively	Until bowel function returned or POD5	No	20	46.1 [14.3]	1‒2	80	Open Colon Resection
	IV Lidocaine: Lidocaine 1‒3 mg.min^-1^.	Post-induction	Until bowel function returned or POD5	No	22	51.2 [17.5]	1‒3	45	
Kuo et al. 2006	TEA: Placed between the 6‒12^th^ T. ‒ Lidocaine 2 mg.kg^-1^ for 10-min, then 3 mg.kg^-1^.hr^-1^.	30-min pre-surgery	Until end of surgery	Yes	20	62.4 [7.4]	‒	55	Colon Cancer Resection
	IV Lidocaine: Lidocaine 2 mg.kg^-1^ for 10 min, then 3 mg.kg^-1^.hr^-1^.	30-min pre-surgery	Until end of surgery	Yes	20	62.8 [6.6]	‒	50	
Jayaprabhu et al. 2022	TEA: Placed between the 8‒10^th^ T. ‒ BUPI 0.25% bolus (3‒5 mL), then 5‒8 mL.hr^-1^ intraoperatively.	Pre-induction	Until POD2	Yes	16	51.1 [12.4]	1‒2	48.1	Laparoscopic Left- Sided Colon and Sphincter-Sparing Rectal Resection
	PACU: 0.1% BUPI + fentanyl 2 mcg.mL^-1^ at 4‒5 mL.hr^-1^.								
	IV Lidocaine: Lidocaine 2 mg.kg^-1^ bolus, then 3 mg.kg^-1^.hr^-1^	Pre-induction	Until 30 mins in PACU	Yes	19	47.7 [15.8]	1‒2	50	
Casas-Arroyave et al. 2023	TEA: Placed between the 6‒10^th^ T. ‒ BUPI 0.1% + M 20 mcg.mL^-1^ at 7 mL.hr^-1^;	Intraoperative	Until POD3	Yes	104	61.8 [15]	2‒3	68.8	Open major abdominal surgery
	IV Lidocaine: Lidocaine 1.5 mg.kg^-1^ bolus, then 1 mg.kg^-1^.hr^-1^	Induction	Until POD1	Yes	106	60 [16.1]	1‒3	68.4	
Wongyingsinn et al. 2010	TEA: Placed between the 8‒9^th^ T. ‒ Lidocaine 2% (3 mL) and BUPI 0.25% bolus (5‒10 mL), then BUPI 5‒8 mL.hr^-1^ intraoperatively.	Pre-induction	Until POD2	No	30	61 [15]	1‒3	63.4	Elective Laparoscopic Colorectal Surgery
	PACU: BUPI 0.1% + M 0.02 mg.mL^-1^								
	IV Lidocaine: Lidocaine 1.5 mg.kg^-1^ bolus, then 2 mg.kg^-1^.hr^-1^ intraoperatively.	Pre-induction	Until POD2	Yes	30	58 [16]	1‒3	63.4	
	PACU: Lidocaine 1 mg.kg^-1^.hr^-1^								
Yazici et al. 2021	TEA: Placed between the 9‒12^th^ T; PCEA with epidural BUPI.	Postoperatively	Until POD1	Yes	25	57 [3]	1‒3	0	Major Oncologic Gynecological surgery
	IV Lidocaine: Lidocaine 1.5 mg.kg^-1^ bolus, then 1.5 mg.kg^-1^.hr^-1^.	Induction	Until POD1	Yes	25	63 [3]	1‒3	0	

Mean [SD]; PCA, Patient-Controlled Analgesia; PCEA, Patient-Controlled Epidural Analgesia; TEA, Thoracic epidural analgesia; T, Thoracic vertebrae; BUPI, Bupivacaine; HM, Hydromorphone; POD, Postoperative Day; IV, Intravenous; PACU, Post-Anesthesia Care Unit; M, Morphine.

### Primary outcomes

We analyzed resting pain scores at 2-, 12-, 24-, 48-, and 72-postoperative hours, and cough pain scores at 24-, 48-, and 72-postoperative hours. Data on cough pain at 1–4 and 12 postoperative hours were unavailable.

We conducted the analysis using mean differences. Two studies assessed pain using the Visual Analog Scale,^17^^,^^19^ two studies used the Numerical Pain Rating Scale,^4^^,^^14^ and two studies employed the Verbal Rating Scale.^13^^,^^18^ All described scales assessed pain on a 0 to 10 scale, with 10 corresponding to the highest levels of pain.

Dynamic pain outcomes were excluded due to limited data. Due to methodological challenges and limitations in the available literature, our analysis focused solely on comparing TEA with intravenous lidocaine. Therefore, our review did not aim to evaluate the non-inferiority of lidocaine.

In all the analyses described, the values presented for the mean difference and confidence intervals refer to lidocaine in comparison with TEA.

### Postoperative pain assessment analysis at rest

Lidocaine was associated with increased pain scores at rest in the first 2 and 24 postoperative hours. (n = 335, MD = 0.72, 95% CI 0.19 to 1.25, p = 0.007423, I^2^ = 83%; n = 402; MD = 0.18, 95% CI 0.12 to 0.23; p < 0.000001, I^2^ = 18%).

At the 48- and 72-postoperative hours, results were not statistically significant (n = 352; MD = 0.13, 95% CI -0.10 to 0.36, p = 0.253343, I^2^ = 0%, PI -0.37 to 0.64; n = 431; MD = -0.01, 95% CI -0.23 to 0.21; p = 0.926858, I^2^ = 0%).

Although 3 studies reported pain scores at the 12 postoperative hour mark, the article by Yazici et al. presented a standard deviation of 0, rendering their data analysis unfeasible. Therefore, this analysis was not conducted.

**Figure 2:**
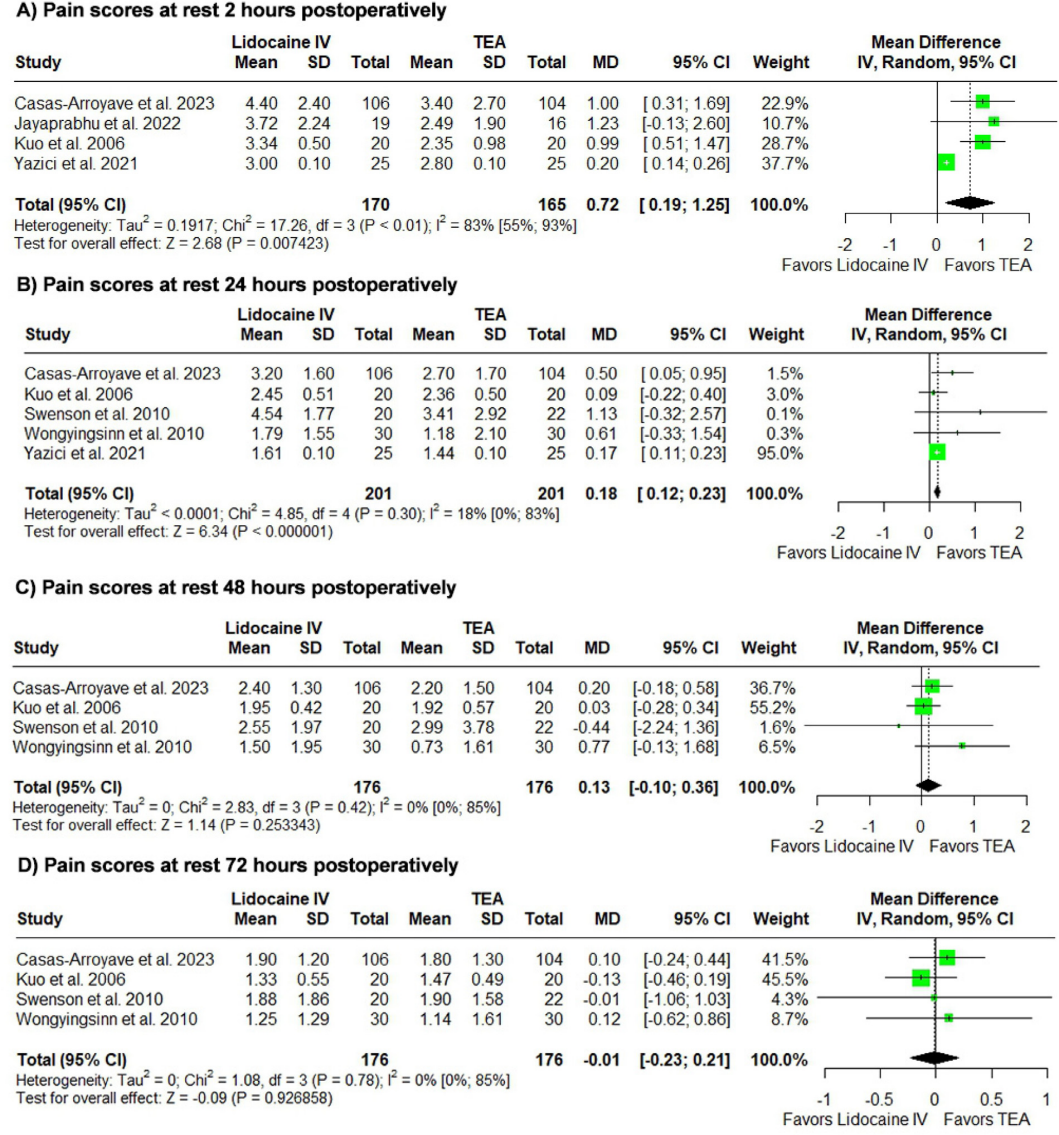
Postoperative pain at rest analysis. Comparison of Pain Scores at Rest Between Perioperative Lidocaine Infusion and Thoracic Epidural Analgesia for Pain Management in Abdominal Surgery.

The corresponding forest plots can be found in Figure 2.

### Postoperative pain assessment analysis on coughing

Lidocaine was associated with increased pain scores on coughing in the 24 postoperative hour mark. (n = 360; MD = 0.36, 95% CI 0.19 to 0.52, p = 0.000019, I^2^ = 2%).

At the 48- and 72-postoperative hours, results were not statistically significant (n = 310; MD = 0.21, 95% CI -0.05 to 0.46; p = 0.109637, I^2^ = 42%; n = 310; MD = 0.03, 95% CI -0.11 to 0.17; p = 0.636252 I^2^ = 0%).

**Figure 3:**
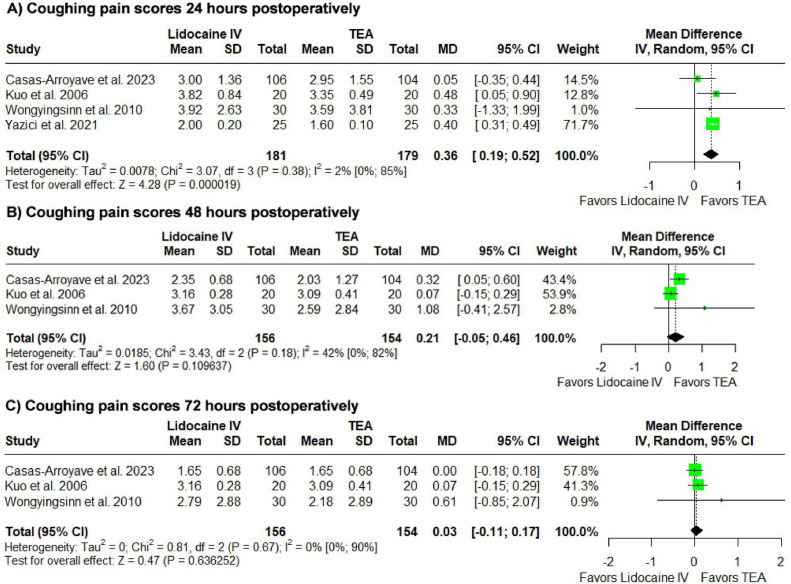
Postoperative pain on coughing analysis. Comparison of Pain Scores on Coughing Between Perioperative Lidocaine Infusion and Thoracic Epidural Analgesia for Pain Management in Abdominal Surgery.

The corresponding forest plots can be found in Figure 3.

### Secondary outcomes

We analyzed time to first flatus, length of hospital stay, and PONV. Results were not statistically significant (flatus: n = 192, MD = 4.25 hours, 95% CI -2.85 to 11.35, p = 0.240993, I^2^ = 83%, Supplementary Figure 1; hospital stay: n = 437; MD = -0.08 days, 95% CI -0.85 to 0.68, p = 0.831266, I^2^ = 68%, Supplementary Figure 2; PONV: n = 362, OR = 0.65, 95% CI 0.40 to 1.07; I^2^ = 0%, Supplementary Figure 3).

The outcomes of morphine consumption at postoperative days-1, -2 and -3, time to advancement to clear diet, time to first bowel movement, and time to first analgesic rescue were not analyzed due to two or fewer studies reporting on them.

### Leave-one-out analysis

We conducted a leave-one-out analysis for outcomes with three or more studies analyzed to assess the robustness of the findings. The results of our leave-one-out analysis for our primary outcomes are described in Table 2.

**Table 2:** Summary of findings.

Comparative Efficacy of Perioperative Lidocaine Infusion Versus Thoracic Epidural Analgesia for Pain Management in Abdominal Surgery: Systematic Review and Meta-analysis						
Patients: Patients undergoing abdominal surgery. Intervention: Perioperative intravenous lidocaine infusion Comparison: Thoracic epidural analgesia						
Outcomes	Relative effect (95% CI)	Prediction intervals (95% PI)	N° of participants (studies)	Certainty of the evidence (GRADE)	Comments^f^	Trial sequential analysis
Pain at rest 2h	MD = 0.72 (0.19 to 1.25)	-1.49 to 2.93	335 (4 RCTs)	⨁◯◯◯ Very low^a^^,^^b^^,^^c^	Omitting Yazici et al. turns I^2^ to 0%. Omitting Casas-arroyave et al. or Kuo et al. renders the result statistically non-significant	Not confirmed by TSA; RIS not achieved
Pain at rest 24h	MD = 0.18 (0.12 to 0.23)	0.09 to 0.26	402 (5 RCTs)	⨁⨁◯◯ Low^c^^,^^d^^,^^e^	Omitting Yazici et al. renders the result statistically non-significant	Confirmed by TSA; RIS not achieved
Pain at rest 48h	MD = 0.15 (-0.06 to 0.36)	-0.37 to 0.64	352 (4 RCTs)	⨁◯◯◯ Very low^a^^,^^c^^,^^e^	The results were consistent and not dependent on any single study	Not confirmed by TSA; RIS not achieved
Pain at rest 72h	MD = -0.01 (-0.23 to 0.21)	-0.49 to 0.47	352 (4 RCTs)	⨁◯◯◯ Very low^a^^,^^c^^,^^e^	The results were consistent and not dependent on any single study	Did not reach the required percentage of the information size necessary for analysis
Pain on cough 24h	MD = 0.36 (0.19 to 0.52)	-0.16 to 0.88	360 (4 RCTs)	⨁◯◯◯ Very low^a^^,^^b^^,^^c^	Omitting Yazici et al. or Kuo et al. renders the result statistically non-significant	Confirmed by TSA; RIS achieved
Pain on cough 48h	MD = 0.21 (-0.05 to 0.46)	-2.17 to 2.59	310 (3 RCTs)	⨁◯◯◯ Very low^a^^,^^c^^,^^e^	Omitting Kuo et al. turns the result statistically significant in favor of TEA and decreases I^2^ to 0%	Not confirmed by TSA; RIS not achieved
Pain on cough 72h	MD = 0.03 (-0.11 to 0.17)	-0.87 to 0.94	310 (3 RCTs)	⨁◯◯◯ Very low^a^^,^^c^^,^^e^	The results were consistent and not dependent on any single study	Did not reach the required percentage of the information size necessary for analysis

The corresponding risk, its 95% Confidence Interval, and its 95% Prediction Intervals were calculated by *R* software. CI, Confidence Interval; PI, Prediction Interval; MD, Mean Difference; TSA, Trial Sequential Analysis; RIS, Required Information Size.

a Downgraded for imprecision due to the fact that the 95% PI around the effect size was large.

b Downgraded once due to high heterogeneity.

c Downgrade once due to high risk of bias.

d Not downgraded for imprecision since the 95% PI and the 95% CI around the effect size are narrow.

e Downgrade once due to despite low statistically low heterogeneity, this low heterogeneity was not confirmed by 95% CI.

f In comments, we describe the results from the leave-one-out analysis. Summary of Findings Table of Perioperative Lidocaine Infusion vs. Thoracic Epidural Analgesia for Pain Management in Abdominal Surgery.

The omission of Yazici et al.^19^ turned the result from time to first flatus statistically significant in favor of TEA (MD = 7.37, 95% CI 1.21 to 13.53, I^2^ = 54%). The results from the length of hospital stay analysis were consistent and not dependent on any single study. The omission of Swenson et al.^18^ turned the result from PONV analysis statistically significant in favor of intravenous lidocaine (OR = 0.59, 95% CI 0.35 to 0.99, I^2^ = 0%).

### Trial sequential analysis

Statistically significant results were deemed confirmed only if they crossed the sequential monitoring boundaries, favoring lidocaine when above the superior monitoring boundary or TEA when below the inferior monitoring boundary. Statistically significant results that did not surpass the sequential monitoring boundaries were not considered confirmed. Additionally, we reported whether the required information size was achieved. None of the analyses reached the futility boundary. Outcomes that did not reach the required percentage of the information size necessary for analysis were identified. A detailed description of the primary outcome analyses is presented in Table 2.

Concerning our secondary outcomes, none of the analyzed outcomes were deemed confirmed, nor did they reach the required information size or reach the futility boundary.

The corresponding images for each trial sequential analysis are available in the Supplementary Material.

### Risk of bias assessment

Two trials exhibited a low overall level of bias.^13^^,^^17^ Two trials exhibited some concerns overall.^14^^,^^18^ Two trials exhibited a high overall risk of bias.^4^^,^^19^

Casas-Arroyave et al.^4^ presented a low risk of bias in all domains analyzed by the RoB2, yet presented serious methodological flaws, which might impair how reliable their results are. However, their study exhibited serious methodological flaws that could compromise the reliability of their findings. Specifically, it was reported that some patients did not understand how to use the PCA, and there was no indication that patients were adequately instructed on its use. This may suggest that some patients requested less analgesia than they actually needed.

Kutay et al.^19^ did not adequately estimate the effect of assignment to intervention, as patients excluded in the post-randomization process were not included in the final analysis.

The image corresponding to the bias analysis is available in Supplementary Figures 4 and 5.

### Funding bias

Jayaprabhu declared no funding.^14^ Kuo et al.,^17^ Wongyingsinn et al.,^13^ and Casas-Arroyave et al.^4^ were funded by governmental agencies or university research funds. Swenson et al.^18^ and Kutay et al.^19^ did not disclose information about funding. There is no evidence suggesting that our analysis is influenced by funding bias.

### Summary of findings

We present an evidence profile table (Table 3) and a summary of findings table (Table 2), both of which assess the quality of evidence using the GRADE criteria.

**Table 3:** GRADE Evidence profile of primary outcomes.

Outcomes	Limitations	Inconsistency/ Heterogeneity	Indirectness	Imprecision	Publication bias	Relative effect (95% CI)	N° of participants (studies)	Certainty of the evidence (GRADE)
Pain at rest 2h	High risk of bias	High I^2^ statistic (95% CI I^2^ 64% to 94%)	Different pain scores scales	Potential imprecision	Not suspected	MD = 0.72 (0.19 to 1.25)	335 (4 RCTs)	⨁◯◯◯ Very low
Pain at rest 24h	High risk of bias	Low I^2^ statistic. Not confirmed (95% CI I^2^ 0% to 83%)	Different pain scores scales	Not suspected	Not suspected	MD = 0.18 (0.12 to 0.23)	402 (5 RCTs)	⨁⨁◯◯ Low
Pain at rest 48h	High risk of bias	Low I^2^ statistic. Not confirmed (95% CI I^2^ 0% to 85%)	Different pain scores scales	Potential imprecision	Not suspected	MD = 0.15 (-0.06 to 0.36)	352 (4 RCTs)	⨁◯◯◯ Very low
Pain at rest 72h	High risk of bias	Low I^2^ statistic. Not confirmed (95% CI I^2^ 0% to 85%)	Different pain scores scales	Potential imprecision	Not suspected	MD = -0.01 (-0.23 to 0.21)	352 (4 RCTs)	⨁◯◯◯ Very low
Pain on cough 24h	High risk of bias	High I^2^ statistic (95% CI I2 83% to 96%)	Different pain scores scales	Potential imprecision	Not suspected	MD = 0.36 (0.19 to 0.52)	360 (4 RCTs)	⨁◯◯◯ Very low
Pain on cough 48h	High risk of bias	Low I^2^ statistic. Not confirmed (95% CI I^2^ 0% to 90%)	Different pain scores scales	Potential imprecision	Not suspected	MD = 0.21 (-0.05 to 0.46)	310 (3 RCTs)	⨁◯◯◯ Very low
Pain on cough 72h	High risk of bias	Low I^2^ statistic. Not confirmed (95% CI I^2^ 0% to 90%)	Different pain scores scales	Potential imprecision	Not suspected	MD = 0.03 (-0.11 to 0.17)	310 (3 RCTs)	⨁◯◯◯ Very low

Evidence Profile Table of Perioperative Lidocaine Infusion vs. Thoracic Epidural Analgesia for Pain Management in Abdominal Surgery.

## Discussion

This study presents a systematic review and meta-analysis comparing the efficacy of TEA versus intravenous lidocaine for postoperative pain management. The main findings on pain scores were as follows: 1) TEA provided superior analgesia with lower pain scores at the 2-hour mark for pain at rest; 2) TEA provided slightly superior analgesia at the 24-hour mark for both pain at rest and during coughing; these were the only analyses confirmed by trial sequential analysis; 3) No statistically significant differences in pain scores were observed at the 48- and 72-hour time points. Regarding the secondary outcomes evaluated, no statistically significant differences were found, including time to first flatus, length of hospital stay, and postoperative nausea and vomiting. The lack of statistically significant differences for certain outcomes should not be interpreted as equivalence between the interventions but rather as inconclusiveness, likely reflecting limitations in statistical power, variability in study design, population heterogeneity, or methodological inconsistencies among the included studies.

These results differ from those of the previous meta-analysis conducted by Weibel et al., which found no differences in pain scores at the evaluated time points. The prior analysis was markedly limited in statistical power, as it included only 102 patients from two randomized clinical trials in the subgroup comparing lidocaine and TEA.^5^ In contrast, all pain score analyses in the present study included a minimum of 300 patients, ensuring greater robustness and allowing for the identification of statistically significant results, with some confirmed through trial sequential analysis. Of the six randomized clinical trials included in our analysis, three were published after the meta-analysis by Weibel et al., highlighting ongoing efforts to identify effective alternatives to thoracic epidural analgesia in abdominal surgeries. Thus, our analysis provides a novel contribution to the existing literature by demonstrating that TEA offers slightly superior analgesia compared to lidocaine on the first postoperative day, whereas no statistically significant differences were observed on the second and third postoperative days. This suggests that the overall difference in analgesic efficacy between these interventions is minimal. These findings reinforce the potential viability of intravenous lidocaine as an alternative to TEA for postoperative pain management and underscore the importance of further studies to determine its clinical significance.

Discussions remain regarding potential alternatives to TEA, particularly strategies that, while statistically inferior, may not demonstrate clinically relevant differences when considering the perioperative management proposed by the ERAS protocol.^3^ Additionally, the analgesic benefits of TEA do not necessarily accelerate postoperative recovery in either open or laparoscopic surgeries and may even prolong hospital stay in the context of laparoscopic procedures.^20^, ^21^, ^22^, ^23^, ^24^, ^25^ In this context, lidocaine may represent a viable alternative, as no statistically significant differences in pain scores were observed at the 48- and 72-hour time points. This suggests that with proper perioperative management, particularly focusing on the first postoperative day, the choice of analgesic technique may be guided by the availability and ease of implementation within individual practices and healthcare settings.

Besides the randomized controlled trials included in this analysis, observational studies offer valuable supplementary data on the comparison between TEA and intravenous lidocaine. Tejedor et al. evaluated these interventions in cytoreductive surgery, and found that, although lidocaine infusion was associated with higher opioid consumption compared to TEA, the level of analgesia provided was similar.^26^ Taking a different approach, Terkawi et al. investigated these two interventions in major abdominal surgeries, where TEA included opioids as part of the regimen. In this case, lidocaine infusion provided comparable analgesia and was also associated with reduced opioid consumption.^27^ Although not included in our systematic review, these observational studies contribute valuable insights into the comparison between TEA and lidocaine infusion, particularly regarding their effects on opioid consumption.

The study by Casas-Arroyave et al., included in this review, analyzed the non-inferiority of intravenous lidocaine compared to TEA in patients undergoing open abdominal surgeries.^4^ They defined non-inferiority as achieving a non-inferiority margin of less than or equal to 1 for dynamic pain scores. The study concluded that intravenous lidocaine was non-inferior to TEA for dynamic pain scores at 24-hours. However, some authors underscore factors that should be considered in the analysis of the study by Casas-Arroyave et al. Coppens et al. pointed out that despite Casas-Arroyave et al. reporting the non-inferiority of intravenous lidocaine, the dynamic pain scale's prespecified non-inferiority margin was exceeded by the 95% CI, and patients receiving intravenous lidocaine analgesia required a greater number of rescue analgesics. This suggests a clinically significant difference in analgesia favoring TEA.^28^ Banik et al., however, highlighted the low failure rate for epidural catheter placement in the Casas-Arroyave et al. study (0.9%) as a potential bias, noting that studies by Leurcharusmee et al., Arnuntasupakul et al. and Hermanides et al., employing similar techniques, observed failure rates ranging from 23% to 32%.^29^, ^30^, ^31^, ^32^

Regarding safety-related outcomes, no statistically significant difference was identified between the interventions in the limited variables that could be analyzed. Complications related to epidural catheters are specific to epidural analgesia; thus, the possibility of failure should be considered when choosing a postoperative analgesia strategy. For instance, in the included studies, epidural analgesia was not administered to one patient (0.9%) in the study by Casas-Arroyave et al.^4^ due to difficulty in advancing the epidural catheter, one patient (5%) in the study by Swenson et al.^18^ experienced epidural catheter dislodgement, and one patient (4%) in the study by Yazici et al.^19^ had their epidural catheter accidentally removed.

This study is subject to some limitations. First, there is considerable variability in the application of systemic lidocaine across the included studies, encompassing differences in dosage and duration of infusion, ranging from the end of surgery to several days postoperatively. Furthermore, clinical heterogeneity ‒ including variations in the type of abdominal surgery ‒ may have influenced our analysis. Additionally, the high risk of bias in certain studies poses a further limitation. While some outcomes lacked sufficient statistical power to detect differences, this should not be interpreted as evidence that a difference would necessarily emerge with a larger sample size. The absence of statistically significant findings must be interpreted cautiously, as it reflects the available data and should not imply a predisposition toward a specific effect. Lastly, our results were assessed as having low to very low certainty of evidence based on the GRADE evaluation.

Nevertheless, our study offers valuable insights for future research. In forthcoming studies comparing the efficacy of these two interventions, it is crucial to provide comprehensive details on epidural placement and to assess the failure rate using verification methods such as epidural waveform analysis, as utilized by Arnuntasupakul et al.^31^ and Leurcharusmee et al.^32^ Moreover, assessing lidocaine levels is essential due to the diverse strategies described for perioperative lidocaine infusion in the literature, as the effectiveness of analgesia may depend on the chosen strategy. Complications specifically associated with each type of analgesia utilized should be thoroughly examined, including issues related to catheterization in epidural analgesia and events of systemic toxicity associated with the intravenous use of lidocaine. Finally, consistent reporting of dynamic pain scores and standardization of lidocaine infusion protocols should be implemented.

Regarding clinical implications, our results suggest the need to optimize analgesia on the first postoperative day for patients undergoing abdominal surgeries with lidocaine infusion to achieve pain relief comparable to that provided by TEA. It is also crucial to recognize that some patients may have contraindications to TEA, underscoring the importance of alternative strategies such as lidocaine infusion.

## Conclusion

TEA provides superior pain control in the early postoperative period, while intravenous lidocaine achieves comparable analgesia after the first day. These findings hold particular relevance within ERAS protocols, in which a balance between optimal analgesia and early postoperative recovery is essential. The choice between TEA and intravenous lidocaine should be guided by clinical circumstances, patient-specific factors, and institutional resources. Future research should standardize lidocaine infusion protocols, rigorously assess epidural placement, and consistently report dynamic pain outcomes to enhance postoperative pain management.

## Authors’ contributions

The authors confirm contribution to the paper as follows:

Conceptualization: Gustavo Roberto Minetto Wegner; Formal analysis: Gustavo Roberto Minetto Wegner, Bruno Franscisco Minetto Wegner, Ramon Huntermann, Francisco José Lucena Bezerra; Methodology: Gustavo Roberto Minetto Wegner, Bruno Franscisco Minetto Wegner, Francisco José Lucena Bezerra; Writing-original draft: Manoela Lenzi Pinto, Júlia Azedias Pessoa Vieira, Amanda Palha de Souza, Francisco José Lucena Bezerra. All authors approved the final version.

## Conflicts of interest

The authors declare no conflicts of interest.

## Appendix Supplementary materials

Image, application 1

## Footnotes

Supplementary material associated with this article can be found in the online version at doi:10.1016/j.bjane.2025.844616.

## References

[1] Levy B.F., Scott M.J., Fawcett W., Fry C., Rockall TA. (2011). Randomized clinical trial of epidural, spinal or patient-controlled analgesia for patients undergoing laparoscopic colorectal surgery. Br J Surg.

[2] Moore L. (2011). Perioperative intravenous lidocaine infusion for postoperative pain control: a meta-analysis of randomized controlled trials. Can J Anaesth.

[3] Wagemans M.F., Scholten W.K., Hollmann M.W., Kuipers AH. (2020). Epidural anesthesia is no longer the standard of care in abdominal surgery with ERAS. What are the alternatives?. Minerva Anestesiol.

[4] Casas-Arroyave F.D., Osorno-Upegui S.C., MA Zamudio-Burbano (2023). Therapeutic efficacy of intravenous lidocaine infusion compared with thoracic epidural analgesia in major abdominal surgery: a noninferiority randomised clinical trial. Br J Anaesth.

[5] Weibel S., Jelting Y., Pace N.L. (2018). Continuous intravenous perioperative lidocaine infusion for postoperative pain and recovery in adults. Cochrane Database Syst Rev.

[6] Cochrane Handbook for Systematic Reviews of Interventions [Internet]. [cited 2022 Jul 17]. Available from: https://training.cochrane.org/handbook.

[7] Page M.J., McKenzie J.E., Bossuyt P.M. (2021). The PRISMA 2020 statement: an updated guideline for reporting systematic reviews. BMJ.

[8] D'Souza R.S., Barrington M.J., Sen A., Mascha E.J., Kelley G.A. (2024). Systematic Reviews and Meta-analyses in Regional Anesthesia and Pain Medicine (Part II): Guidelines for Performing the Systematic Review. Anesth Analg.

[9] Ouzzani M., Hammady H., Fedorowicz Z., Elmagarmid A. (2016). Rayyan ‒ a web and mobile app for systematic reviews. Syst Rev.

[10] Wan X., Wang W., Liu J., Tong T. (2014). Estimating the sample mean and standard deviation from the sample size, median, range and/or interquartile range. BMC Med Res Methodol.

[11] Luo D., Wan X., Liu J., Tong T. (2018). Optimally estimating the sample mean from the sample size, median, mid-range, and/or mid-quartile range. Stat Methods Med Res.

[12] WebPlotDigitizer - Copyright 2010-2024 Ankit Rohatgi [Internet]. [cited 2024 Apr 24]. Available from: https://apps.automeris.io/wpd/.

[13] Wongyingsinn M., Baldini G., Charlebois P., Liberman S., Stein B., Carli F. (2011). Intravenous lidocaine versus thoracic epidural analgesia: a randomized controlled trial in patients undergoing laparoscopic colorectal surgery using an enhanced recovery program. Reg Anesth Pain Med.

[14] Jayaprabhu N.B., Avula J., Chandy T.T., Varghese G., Yadav B., Rebekah G. (2022). A Randomized Controlled Trial Comparing Intravenous Lidocaine Infusion With Thoracic Epidural for Perioperative Analgesia and Quality of Recovery After Surgery in Laparoscopic Left-Sided Colon and Sphincter-Sparing Rectal Resection Surgery. Cureus.

[15] Goldet G., Howick J. (2013). Understanding GRADE: an introduction. J Evid-Based Med.

[16] Sterne J.A.C., Savović J., Page M.J. (2019). RoB 2: a revised tool for assessing risk of bias in randomised trials. BMJ.

[17] Kuo C.P., Jao S.W., Chen K.M. (2006). Comparison of the effects of thoracic epidural analgesia and i.v. infusion with lidocaine on cytokine response, postoperative pain and bowel function in patients undergoing colonic surgery. Br J Anaesth.

[18] Swenson B.R., Gottschalk A., Wells L.T. (2010). Intravenous lidocaine is as effective as epidural bupivacaine in reducing ileus duration, hospital stay, and pain after open colon resection: a randomized clinical trial. Reg Anesth Pain Med.

[19] Kutay Yazici K., Kaya M., Aksu B., Ünver S. (2021). The Effect of Perioperative Lidocaine Infusion on Postoperative Pain and Postsurgical Recovery Parameters in Gynecologic Cancer Surgery. Clin J Pain.

[20] Irani J.L., Hedrick T.L., Miller T.E. (2023). Clinical Practice Guidelines for Enhanced Recovery After Colon and Rectal Surgery From the American Society of Colon and Rectal Surgeons and the Society of American Gastrointestinal and Endoscopic Surgeons. Dis Colon Rectum.

[21] Al-Mazrou A.M., Kiely J.M., Kiran RP. (2019). Epidural analgesia in the era of enhanced recovery: time to rethink its use?. Surg Endosc.

[22] Torgeson M., Kileny J., Pfeifer C., Narkiewicz L., Obi S. (2018). Conventional Epidural vs Transversus Abdominis Plane Block with Liposomal Bupivacaine: A Randomized Trial in Colorectal Surgery. J Am Coll Surg.

[23] Halabi W.J., Kang C.Y., Nguyen V.Q. (2014). Epidural analgesia in laparoscopic colorectal surgery: a nationwide analysis of use and outcomes. JAMA Surg.

[24] Borzellino G., Francis N.K., Chapuis O., Krastinova E., Dyevre V., Genna M. (2016). Role of Epidural Analgesia within an ERAS Program after Laparoscopic Colorectal Surgery: A Review and Meta-Analysis of Randomised Controlled Studies. Surg Res Pract.

[25] Hübner M., Blanc C., Roulin D., Winiker M., Gander S., Demartines N. (2015). Randomized clinical trial on epidural versus patient-controlled analgesia for laparoscopic colorectal surgery within an enhanced recovery pathway. Ann Surg.

[26] Tejedor A., Bijelic L., Polanco M., Pujol E. (2023). Intravenous lidocaine infusion compared to thoracic epidural analgesia in cytoreductive surgery with or without heated intraperitoneal chemotherapy. A retrospective case-cohort study. Eur J Surg Oncol.

[27] Terkawi A.S., Tsang S., Kazemi A. (2016). A Clinical Comparison of Intravenous and Epidural Local Anesthetic for Major Abdominal Surgery. Reg Anesth Pain Med.

[28] Coppens S., Uppal V., Hoogma D.F., Merjavy P., Rex S. (2023). Therapeutic efficacy of intravenous lidocaine infusion compared with thoracic epidural analgesia in major abdominal surgery. Comment on Br J Anaesth.

[29] Banik R.K., Tran B.W., Belfar A., Akhtaruzzaman A.K.M., Nada E., Hanson N. (2023). Therapeutic efficacy of intravenous lidocaine infusion compared with thoracic epidural analgesia in major abdominal surgery: factors affecting successful thoracic epidural analgesia. Comment on Br J Anaesth.

[30] Hermanides J., Hollmann M.W., Stevens M.F., Lirk P. (2012). Failed epidural: causes and management. Br J Anaesth.

[31] Arnuntasupakul V., Van Zundert TCRV, Vijitpavan A. (2016). A Randomized Comparison Between Conventional and Waveform-Confirmed Loss of Resistance for Thoracic Epidural Blocks. Reg Anesth Pain Med.

[32] Leurcharusmee P., Arnuntasupakul V., Chora De La Garza D. (2015). Reliability of Waveform Analysis as an Adjunct to Loss of Resistance for Thoracic Epidural Blocks. Reg Anesth Pain Med.

