# Supplementary material for: Comparative efficacy of perioperative lidocaine infusion versus thoracic epidural analgesia for pain management in abdominal surgery: systematic review and meta-analysis
Source: Braz J Anesthesiol. 2025 Mar 29;75(3):844616. doi: 10.1016/j.bjane.2025.844616 (PMC11999601; doi:10.1016/j.bjane.2025.844616)

**BJAN-D-24-00541**

**Supplementary Material**

**SUPPLEMENTARY MATERIAL**

**1. Literature search strategy**

**2. Supplementary table 1**

**3. Secondary outcomes**

**4. Risk of bias assessment**

**5. Trial sequential analysis**

**1. Literature search strategy**

| Pubmed: |
| --- |
| ("Lidocaine" AND ("Infusion" OR "intravenous" OR "perioperative" OR "intraoperative" OR "Systemic")) AND (("Randomized" OR "Randomised") AND ("Controlled" OR "Clinical") AND ("Trial" OR "Study")) AND "Epidural" |
|  |
| **Embase:** |
| ("Lidocaine" AND ("Infusion" OR "intravenous" OR "perioperative" OR "intraoperative" OR "Systemic")) AND (("Randomized" OR "Randomised") AND ("Controlled" OR "Clinical") AND ("Trial" OR "Study")) AND "Epidural" |
|  |
| **Cochrane library:** |
| ("Lidocaine" AND ("Infusion" OR "intravenous" OR "perioperative" OR "intraoperative" OR "Systemic")) AND (("Randomized" OR "Randomised") AND ("Controlled" OR "Clinical") AND ("Trial" OR "Study")) AND "Epidural" |
|  |
| **Web of science** |
| ("Lidocaine" AND ("Infusion" OR "intravenous" OR "perioperative" OR "intraoperative" OR "Systemic")) AND (("Randomized" OR "Randomised") AND ("Controlled" OR "Clinical") AND ("Trial" OR "Study")) AND "Epidural" |

**2. Supplementary Table 1**

| **Study** | **Intervention** | **PCA/PCEA** | **Drug** | **Dose** | **Lockout** | **Max dose** | **Time of use** |
| --- | --- | --- | --- | --- | --- | --- | --- |
| Kuo et al. 2006 | TEA | PCEA method | Morphine (0.1 mg.mL^-1^) in 100 mL of Ropivacaine 0.2% | 10 mL at first trigger, then 4 mL on demand | 15 minutes | ‒ | 72 hours |
|  | IV Lidocaine | PCEA method | Morphine (0.1 mg.mL^-1^) in 100 mL of Ropivacaine 0.2% | 10 mL at first trigger, then 4 mL on demand | 15 minutes | ‒ | 72 hours |
| Jayaprabhu et al. 2022 | TEA | PCA method | Fentanyl | 20 mcg on demand | 15 minutes | ‒ | 24 hours |
|  | IV Lidocaine | PCA method | Fentanyl | 20 mcg on demand | 15 minutes | ‒ | 24 hours |
| Casas-Arroyave et al. 2023 | TEA | All subjects were given a morphine schedule through a PCA system.^a^ | | | | | |
|  | IV Lidocaine | All subjects were given a morphine schedule through a PCA system.^a^ | | | | | |
| Wongyingsinn et al. 2010 | TEA | Did not use PCA or PCEA | | | | | |
|  | IV Lidocaine | PCA method | Morphine | 1‒2 mg on demand | 7 minutes | ‒ | 48 hours |
| Yazici et al. 2021 | TEA | PCEA method | Bupivacaine 0.125% solution | 4 mL on demand | 20 minutes | ‒ | NR |
|  | IV Lidocaine | PCA method | Morphine 1 mg.mL^-1^ | 1 mg on demand | 10 minutes | 30 mg/4h | NR |

PCA, Patient-Controlled Analgesia; PCEA, Patient-Controlled Epidural Analgesia; TEA, Thoracic Epidural Analgesia; IV, Intravenous.

^a^ Description reproduced exactly as given in the original article, no further details are provided by the article.

**3. Secondary outcomes**

**Supplementary Figure 1** Time to first flatus (hours).


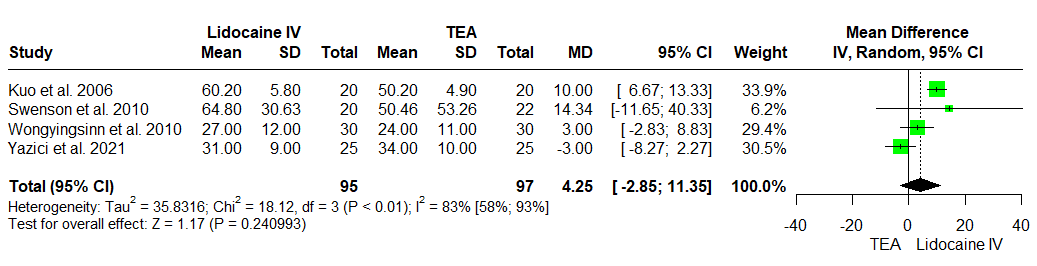


**Supplementary Figure 2** Inpatient time (days).

**
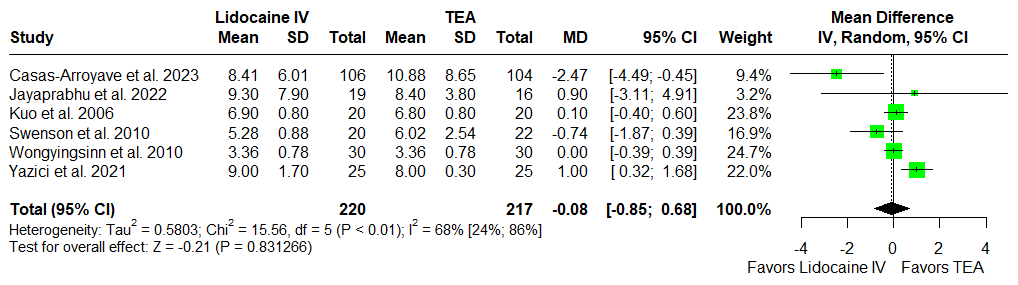
**

**Supplementary Figure 3** PONV.

**
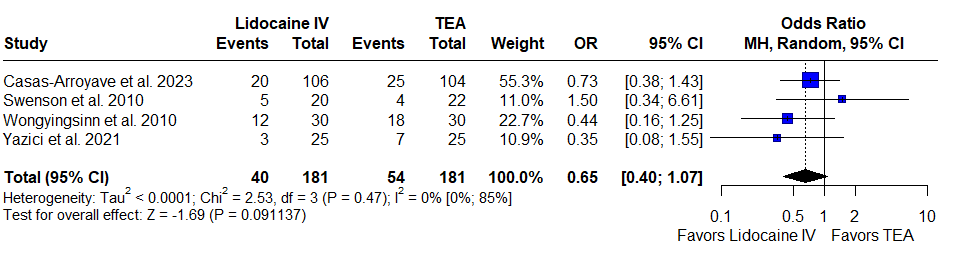
**

**4. Risk of bias assessment**

**Supplementary Figure 4** Risk of bias assessment.


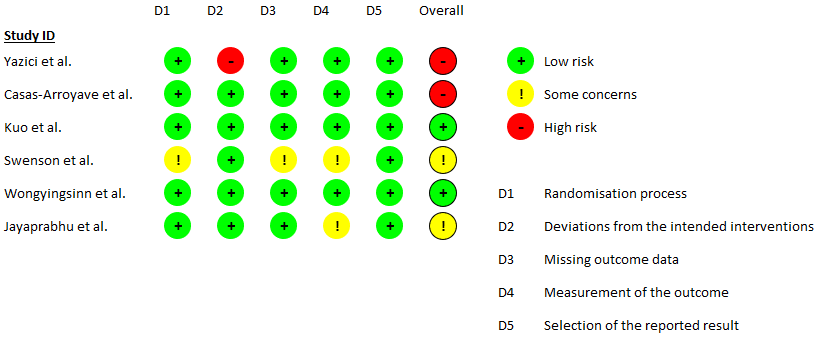


**Supplementary Figure 5** Overall risk of bias.


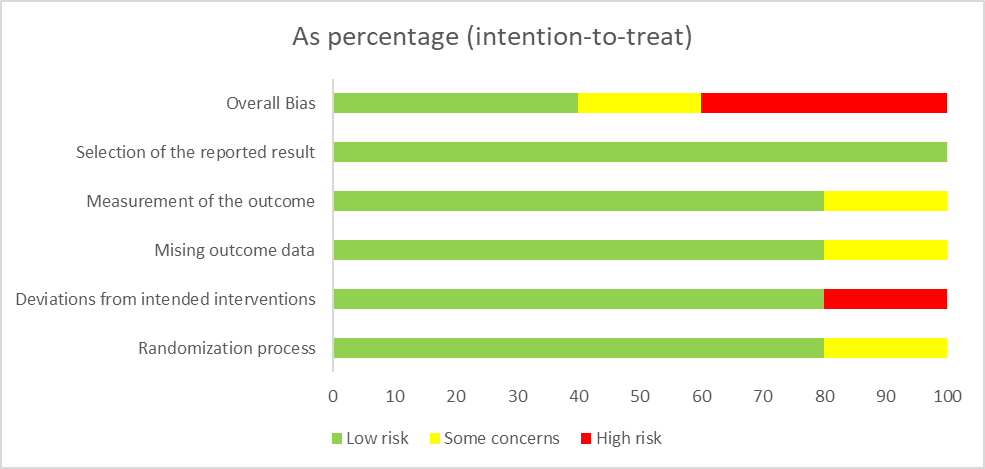


**5. Trial sequential analysis**

**Supplementary Figure 6** Pain scores at rest 2-hours postoperatively.


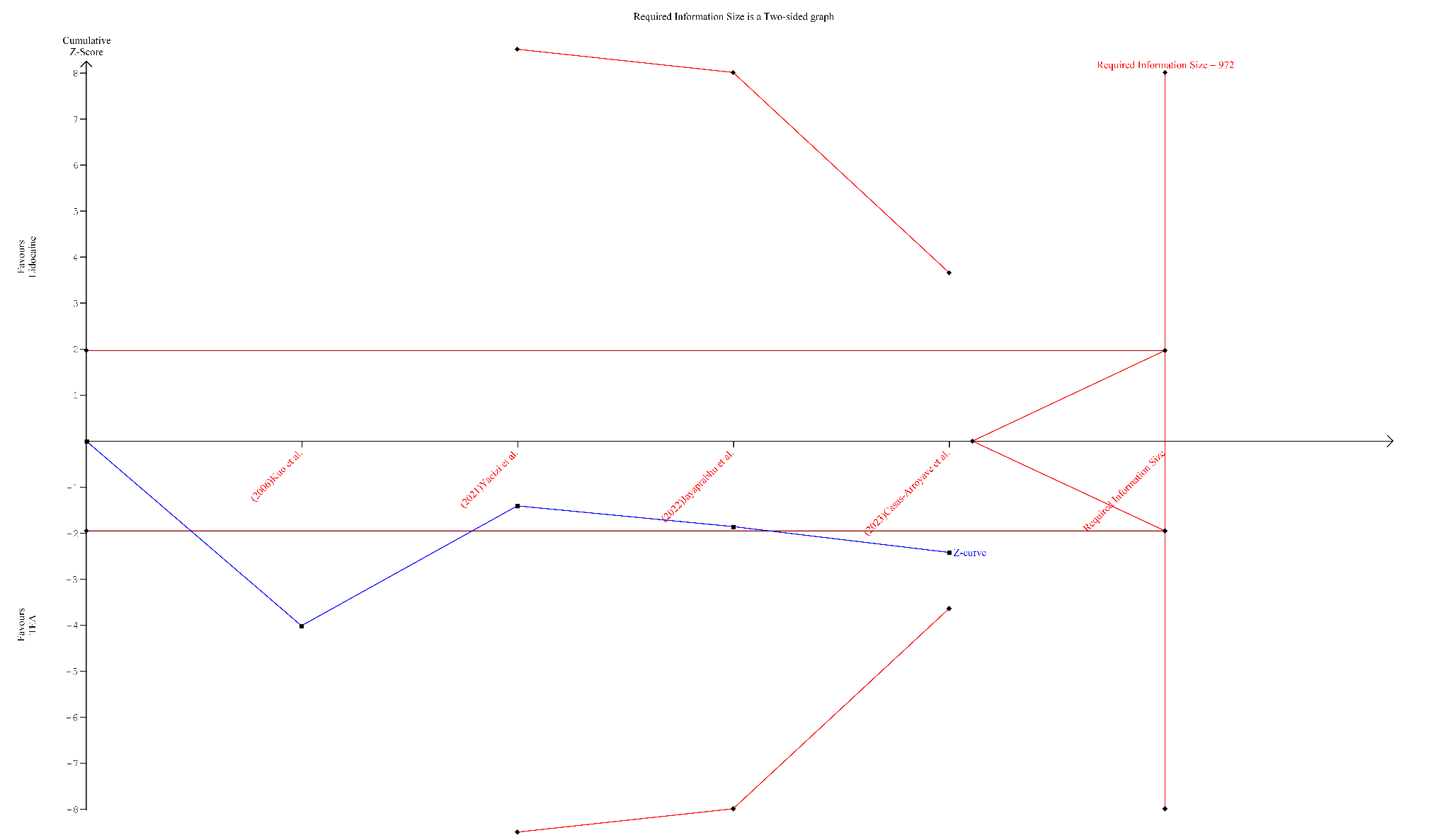


**Supplementary Figure 7** Pain scores at rest 24-hours postoperatively.


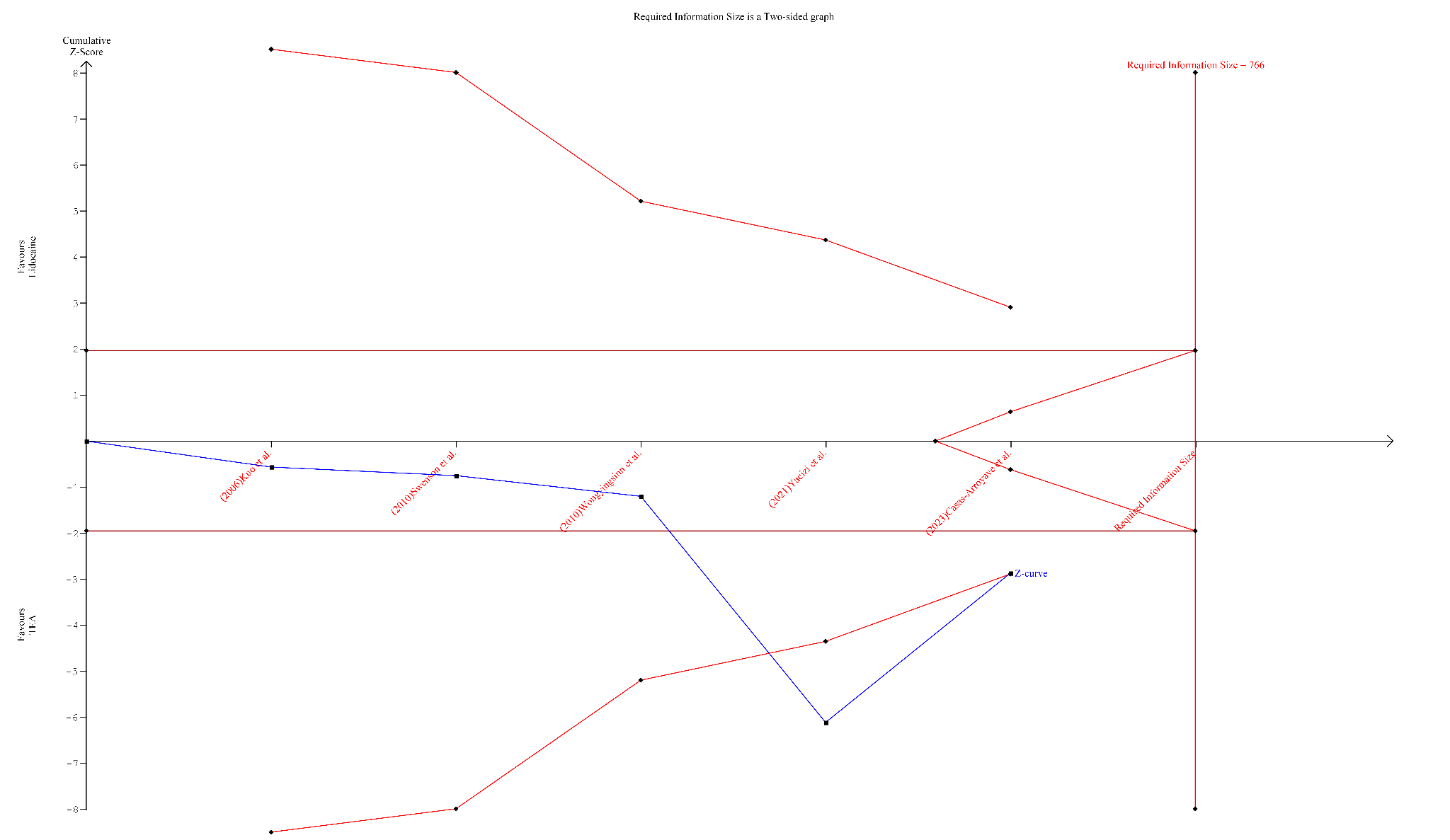


**Supplementary Figure 8** Pain scores at rest 48-hours postoperatively.


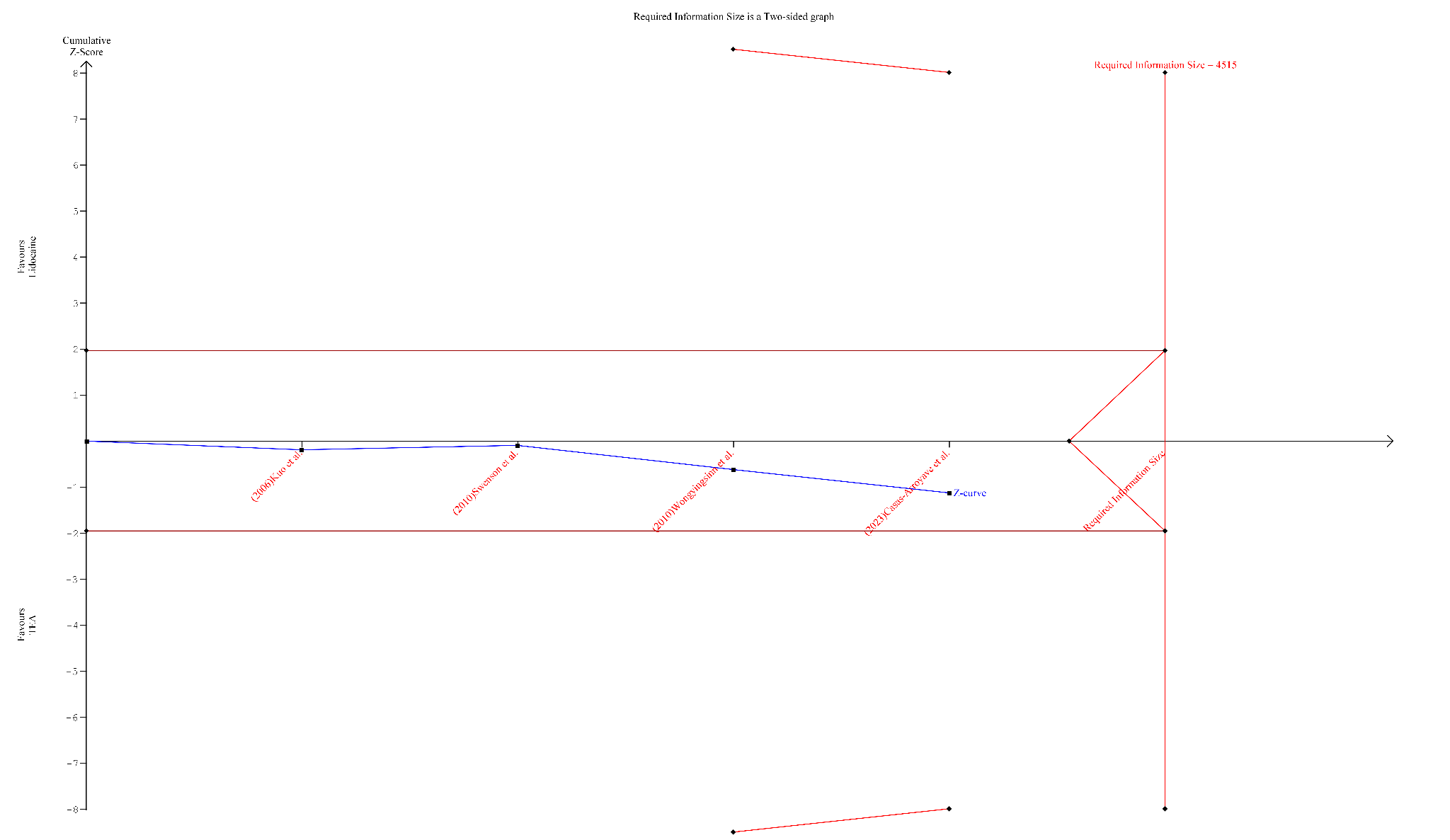


**Pain scores at rest 72-hours postoperatively**

Did not reach the required percentage of the information size necessary for analysis.

**Supplementary Figure 9** Pain scores on coughing 24 hours postoperatively.


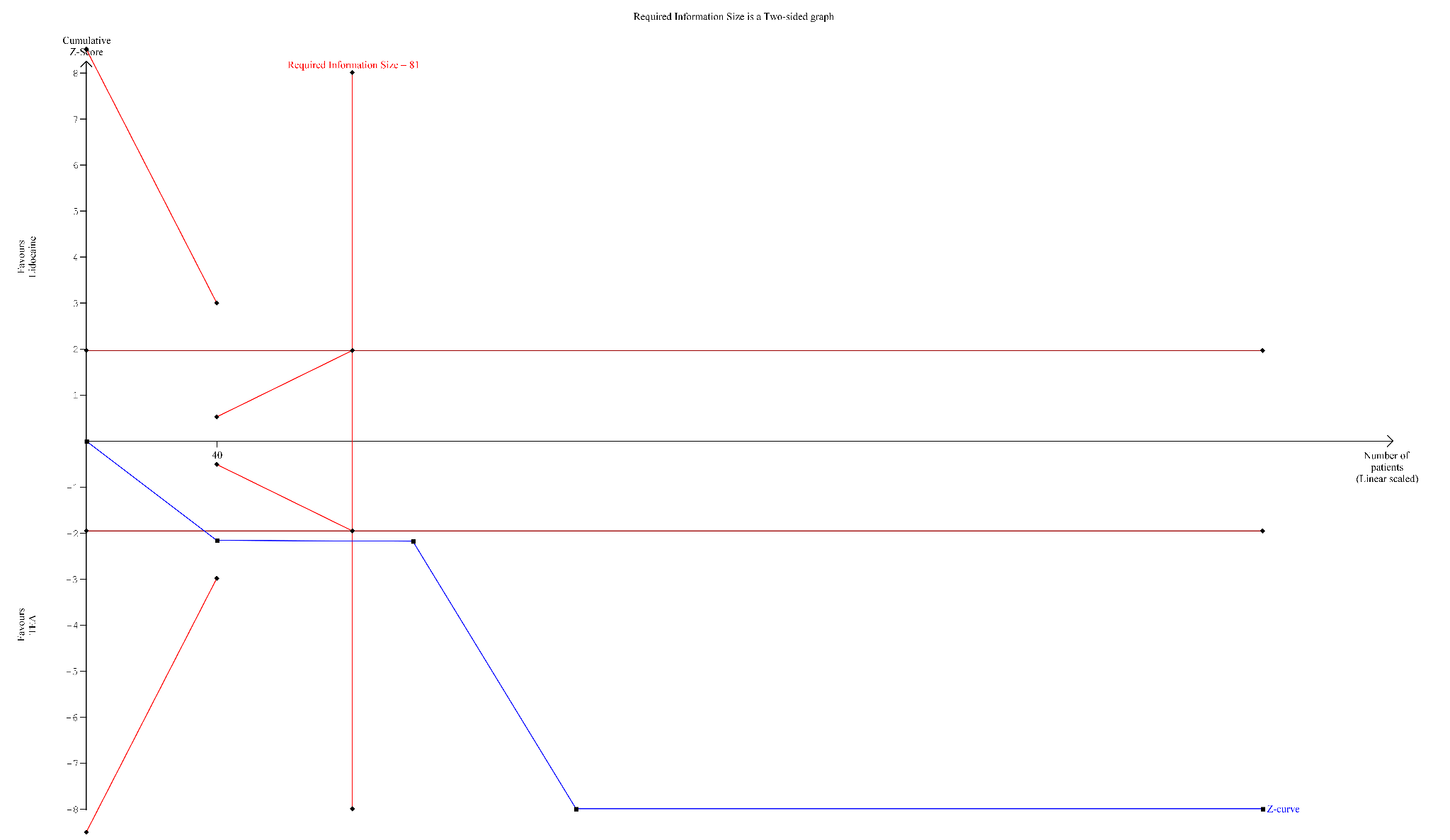


**Supplementary Figure 10** Pain scores on coughing 48 hours postoperatively.


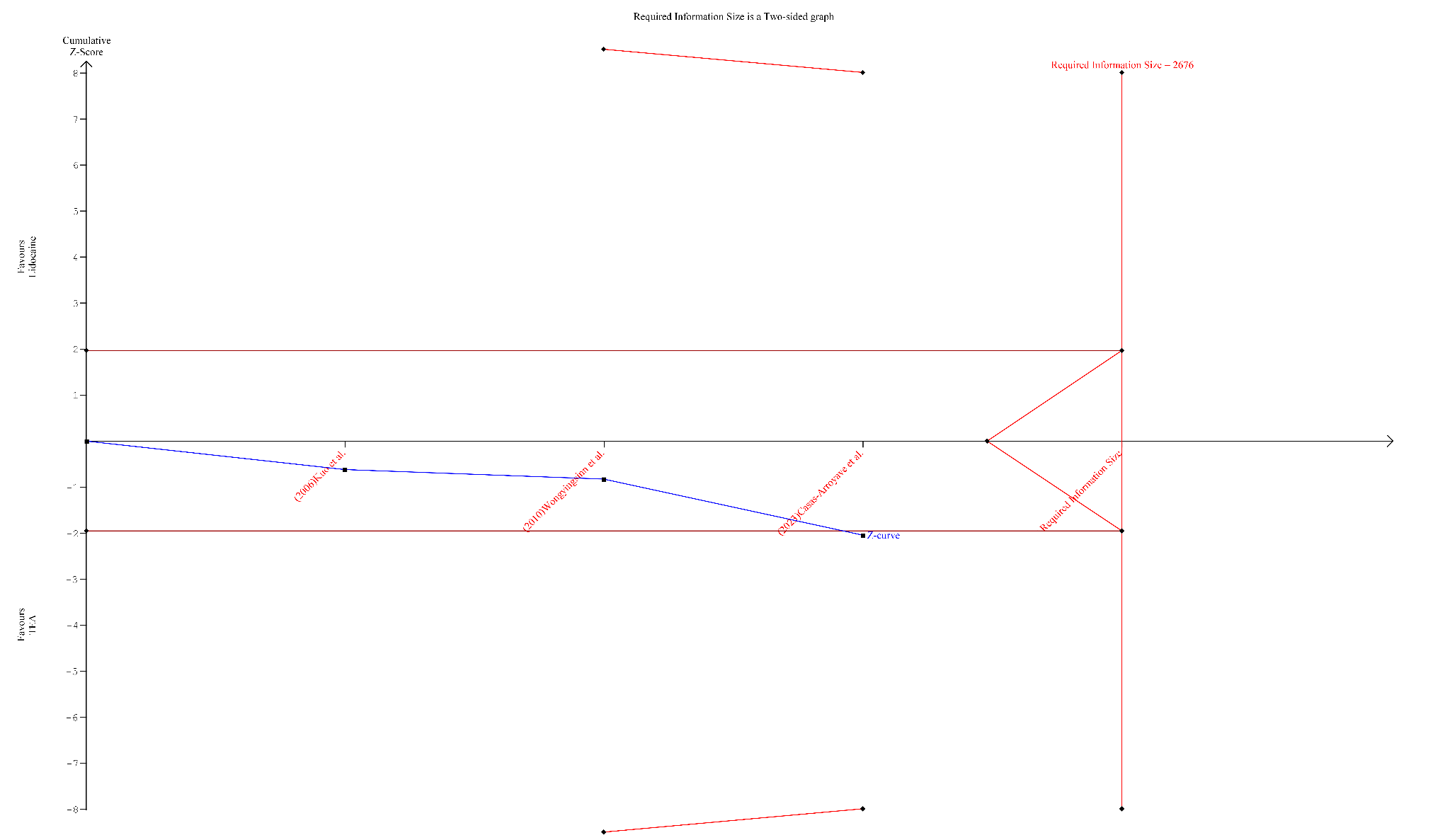


**Pain scores on coughing 72-hours postoperatively**

Did not reach the required percentage of the information size necessary for analysis.

**Supplementary Figure 11** Time to first flatus.


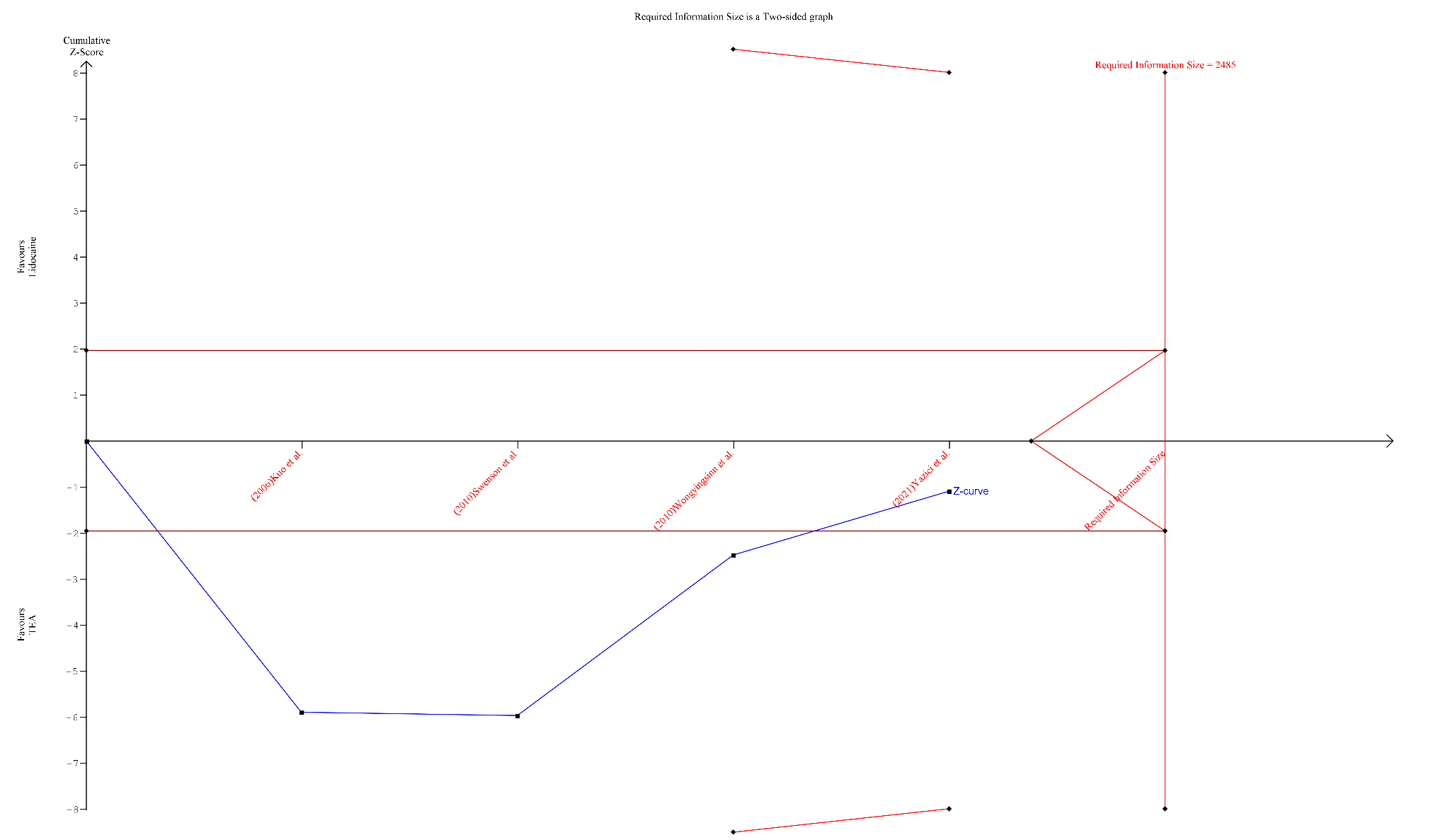


**Length of hospital stay**

Did not reach the required percentage of the information size necessary for analysis.

**Supplementary Figure 12** PONV.


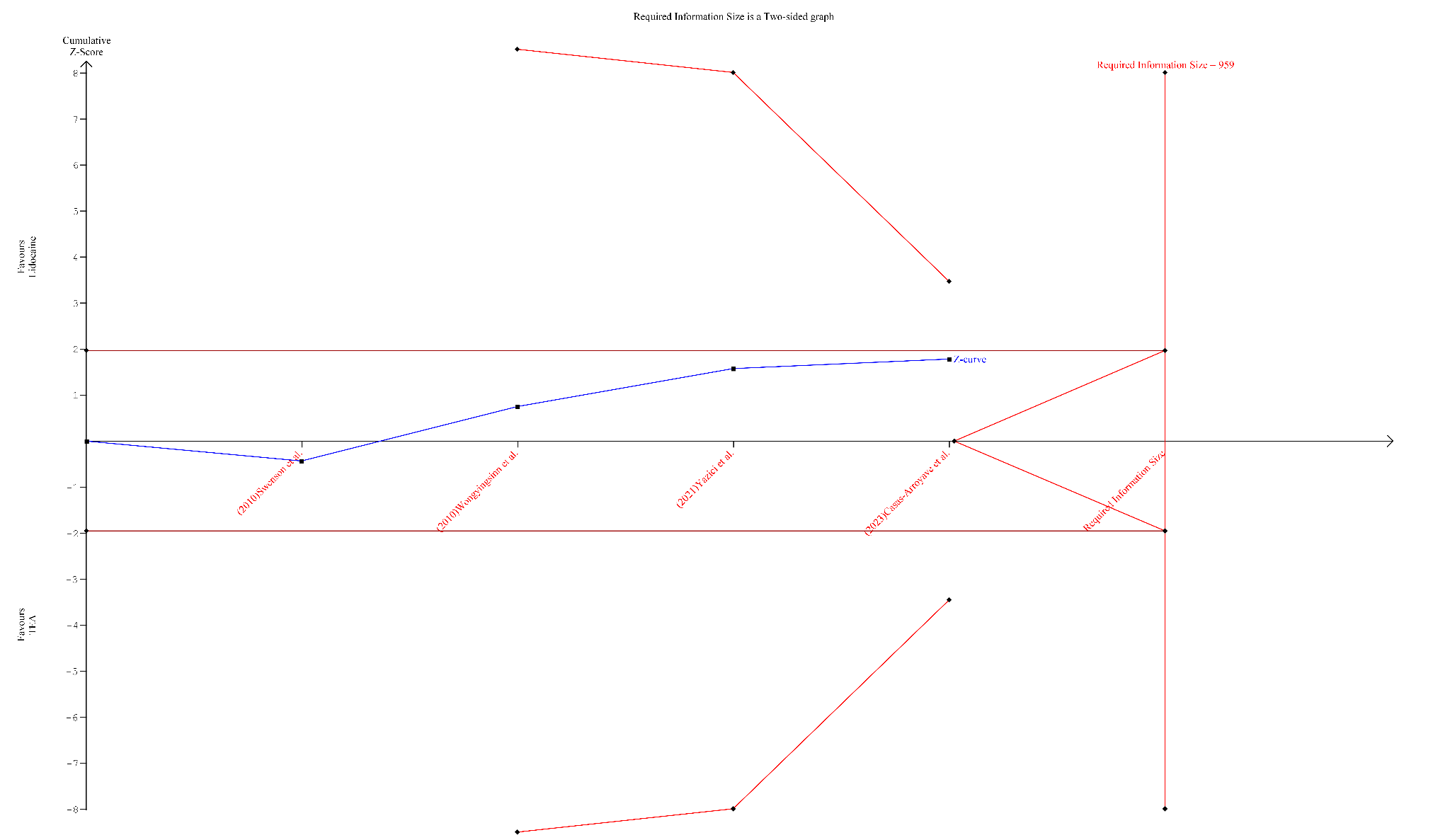

Supplement: Supplementary file 1 [file mmc1.docx]
